# Socio-demographic determinants as predictors of oral hygiene status and gingivitis in schoolchildren aged 7-12 years old: A cross-sectional study

**DOI:** 10.1371/journal.pone.0208886

**Published:** 2018-12-14

**Authors:** Saeed Bashirian, Shabnam Seyedzadeh-Sabounchi, Samane Shirahmadi, Ali-Reza Soltanian, Akram Karimi-shahanjarini, Farshid Vahdatinia

**Affiliations:** 1 Department of Public Health, Health Education and Social Determinants of Health Research Center, Hamadan University of Medical Sciences, Hamadan, Iran; 2 Department of Dental Public Health, School of Dentistry, Hamadan University of Medical Sciences, Hamadan, Iran; 3 Department of Public Health, Hamadan University of Medical Sciences, Hamadan, Iran; 4 Department of Biostatistics, School of Public Health and Modeling of No Communicable Diseases Research Center, Hamadan University of Medical Sciences, Hamadan, Iran; 5 Dental Research Centers, School of Dentistry, Hamadan University of Medical Sciences, Hamadan, Iran; Griffith University, AUSTRALIA

## Abstract

**Objective:**

Gingivitis and poor oral hygiene status are the most prevalent oral diseases among primary school students. Poor oral hygiene status, gingivitis and socio-demographic determinants have been shown to be associated with periodontal diseases. There is limited information on the gingivitis and oral hygiene status among Iranian children. In the present study, the status of gingivitis, oral hygiene status, and their association with socio-demographic determinants among schoolchildren aged 7–12 years old in Hamadan were investigated.

**Methods:**

In this cross-sectional study, 988 primary school students aged 7–12 years old were selected. The oral hygiene status was measured through Simplified Oral Hygiene Index (OHI-S) and Community Periodontal Index (CPI) was used to evaluate gingival bleeding and calculus. CPI was measured using a standardized protocol to investigate gum bleeding and calculus. The oral hygiene was classified as good, fair or poor based on calculus and debris measurements. Age, gender, educational level, occupation and residence district of parents, dental pain experience in the last year and whether parents supervised their children while brushing were assessed by the questionnaires. The collected data were analyzed using descriptive statistics and logistic regression analysis.

**Results:**

The oral hygiene status was good in 644 students (65.20%), fair in 341 (34.50%) and poor in three (0.30%). Moreover, the results of CPI depicted that 639 students (64.07%) had healthy periodontium, 320 (32.40%) had periodontal bleeding and 29 (2.9%) were with calculus.

Higher percentage of the boys in the age group of 12 years old had periodontal bleeding and fewer good oral hygiene. The results of CPI and OHI-S scores depicted that more than half of the primary school students had healthy gums and periodontium (64.1%) and good oral hygiene status (65.2%).

There were significant statistical associations between age and residence district with calculus measured by the CPI, also between gender, age, residence district, and mother's occupation with the gingival bleeding measured by the CPI. Furthermore, age and mother's occupation were significantly associated with OHI-S index.

**Conclusions:**

In general, the periodontal health status is poorer in students attending suburban schools compared to those in urban schools in Hamadan. Since there are significant associations between gender, school districts and mother’s occupation with oral hygiene index among schoolchildren in primary schools, considering them in schools’ oral health program design might be useful.

## Introduction

Oral diseases are important since they can lead to tooth loss and affect the general health and wellbeing of children through influencing their diet, speech and deteriorating already present chronic diseases such as diabetes and heart disease [1]. Furthermore, oral problems and tooth loss may lead to low positive self-image, self-confidence and consequently, low quality of life in children [2]. Although tooth decay has been recognized as the most persistent and frequent childhood dental problem, many children and adults worldwide have symptoms of periodontal diseases [3]. Presence of these types of diseases in childhood can predict future dental problems and affect the growth and developmental process as well as the cognitive functions in children. Most of the periodontal diseases can be reversed in the early stages; however, if they are not treated and progress, they can become painful, irreversible and their complications usually remain for a lifetime [4]. Therefore, gingival and periodontal diseases might eventually lead to tooth loss [5].

It has been demonstrated that occasional tooth brushing and high consumption of sugary foods are associated with increased severity and extent of periodontal inflammation[6]. On the other hand, the periodontal inflammation and bleeding can affect the ability to clean the teeth effectively [7]. Studies in Iran also show that several factors such as inappropriate nutrition, the absence of oral and dental hygiene behaviors and inadequate knowledge of families and children have led to an increase in the prevalence of poor oral hygiene status and gingivitis in children [8–10].In an oral health survey conducted in 2005,6.8% of Iranian children aged 5–6 years old had gingivitis without calculus. Moreover, in 2005, 13.2% of 12-year-old children had gingivitis without calculus [11]. In 2012, the national survey conducted by Iran’s Ministry of Health and Medical Education depicted the incidence of periodontal gingival bleeding 9.7% in children aged 5–6 years old and 26.9% in 12 year old children [12].The results of the same survey indicated that 25.6% of the population in the age group of 35–44 years old had periodontal pockets (4–5 mm) [12].

Since the treatment of periodontal disease is complicated and costly, and specialized services are not available in all parts of the country [13–15], preventive dentistry interventions and services are a necessity in Iran. In addition, the World Health Organization (WHO) suggests that oral healthcare should be performed through regular monitoring of oral health status. Such surveillance activities in each country are conducted by calibrated researchers every 5 to 6 years, and must be carried out in similar communities and environments, and in certain ages and representative age groups using standard criteria for recording clinical conditions [16]. In Iran, limited studies have been recently performed on the gingivitis and oral hygiene status. Since any planning for health and prevention requires the availability of accurate, up-to-date and standard information, it is essential to conduct studies for assessing the periodontal health indicators as well as other factors affecting these indicators among primary school students. The aim of this study was to evaluate the parameters of gingivitis and oral hygiene status in western Iran using the Simplified Oral Hygiene Index (OHI-S) and the Community Periodontal Index (CPI). In addition, due to the lack of evidence on demographic factors affecting OHI-S and CPI in children aged 7–12 years old in Hamadan, the relationship between these indices and the socio-demographic determinants (age, gender, dental pain experience, parental supervision, parental education, and parental occupation and residence districts) were studied among primary school students.

## Materials and methods

### Ethical considerations

The Ethics Committee of Hamadan University of Medical Sciences approved this study (IR.UMSHA.REC.1394.473). The parents signed a written informed consent in which they were explained about the study objectives, the risks and benefits and the voluntary nature of participation in the study. Verbal consent and permission were also obtained from all children aged 7 to 12 years. All de-identified data were collected from the study participants. No direct benefits or rewards were paid to participants for their participation in this study.

### Study population

The current cross-sectional study was conducted on primary school students aged 7 to 12 years old in Hamadan, western Iran, between March and May 2016. Only children whose parents/legal guardians declared their informed consent participated in the study.

### Sampling method

**Fig 1:**
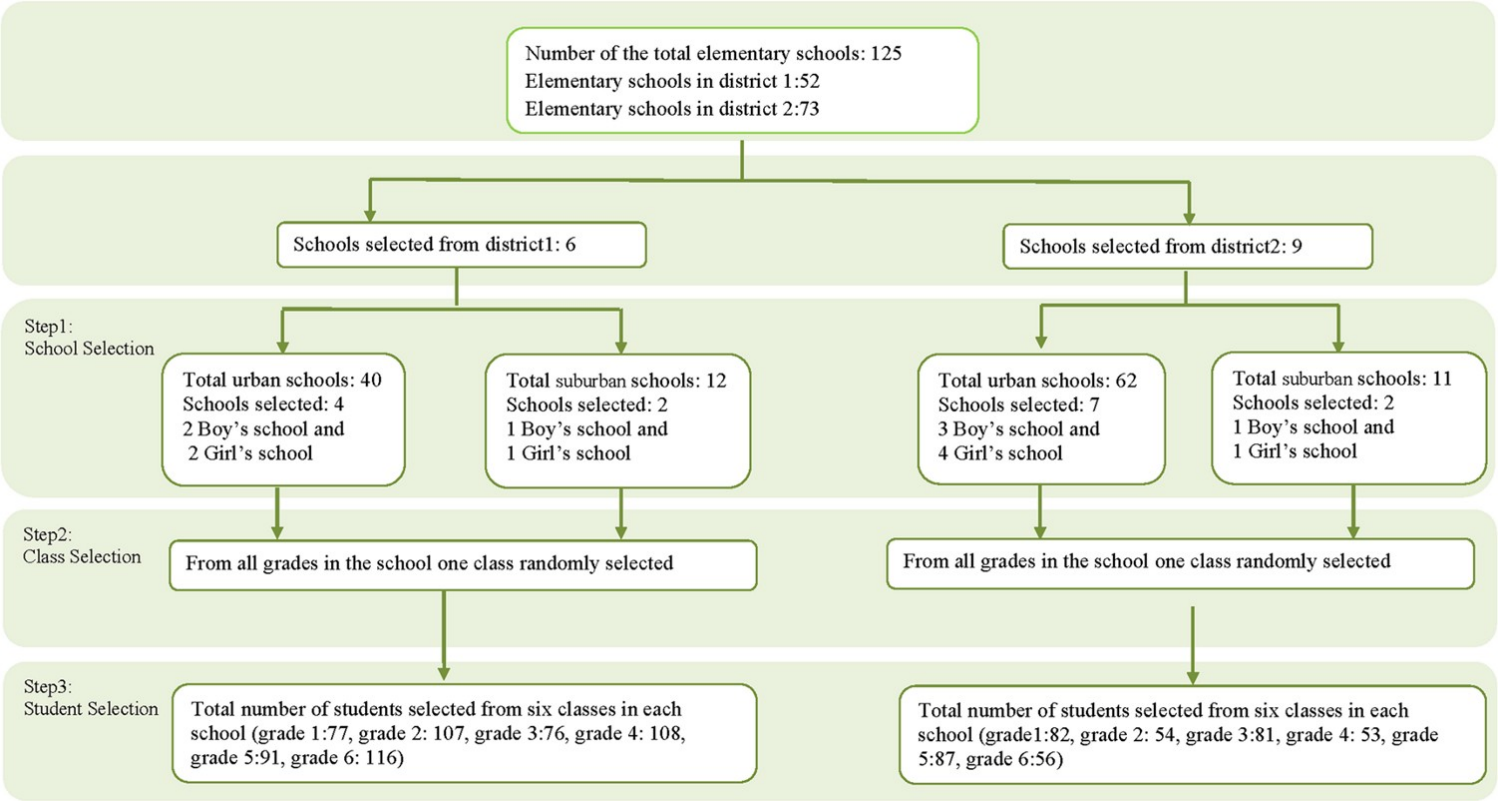
Flowchart of the sampling process and study subject selection among 125 elementary schools.

The sampling method was multistage cluster sampling (at three steps) aimed at selecting eligible participants [17, 18]. The three steps have been shown in Fig 1. Step one involved the random selection of schools from two educational districts (District 1 and 2), proportional to the number of girl and boy schools, and access to health services in each district, urban and suburban. In the step two, classes were randomly selected from all classroom grades available in each primary school chosen at step one. Step three was random selection of primary school student. Only those students who attended classes during the study period and were eligible at the time of the study were enrolled in the study (Fig 1).

The sample size was calculated based on this formula (z^2^_1-α/2_) σ^2^/d^2^ and the standard deviation based on previous studies was inserted as 2.74[19]. The required precision of the estimate (d) was set at 22% (variance was assumed to be 0.03) and the Confidence Interval was 95%, [(1.96)^2^(2.74)^2^/ (0.22)^2^]. Then, the design effect of 1.5 and non-response error of 10% was added. Totally, 988 students were examined.

The study inclusion criteria were the age range of 7–12 years old, no history of systemic diseases, and not being under orthodontic treatment at the time of study. The dental indices were measured by using Community Periodontal Index (CPI) and Simplified Oral Hygiene Index (OHI-S)[20, 21]. Apart from the dental examination of primary school students, they were also interviewed to record their socio-demographic data (including both individual and family characteristics). The questionnaire included age, gender, parental education, parental occupation, the residence district (suburban and urban), history of dental pain experience as well as parental supervision on oral hygiene behavior. Students were asked about dental pain experience through a single question: “Have you experienced toothache in the last twelve months?” They were also asked about parental supervision through a single question: “Do your parent watch or advise you while you brush your teeth?” The recording details of the questionnaire variables have been provided in the study by Bashirian et al.[22]. All questionnaires were completed through interviewing participants by the researcher. Parental occupations and level of education were also reviewed in the schools’ record books. These books are developed for each student and their parents’ occupation in all primary schools. The researcher and examiner made two follow-ups to the schools in addition to the first time visit to avoid possible missing data in the questionnaires and examination.

### Dental examinations

All the dental examinations were performed by a postgraduate dental student experienced in the use of WHO criteria. He was calibrated by dental public health faculty at the School of Dentistry, Hamadan University of Medical Sciences. He had clinically examined more than 500 patients mainly children regarding their dental and oral health in the School of Dentistry, Hamadan University of Medical Sciences. The Kappa agreement between the examiner and dental public health faculty were 0.91 and 0.86 for CPI and OHI-S, respectively.

These examinations were conducted according to infection control standards [23]. The clinical examiner wore disposable gloves, masks and protective glasses. In addition, the dental examination utilities and gloves were changed for each student to reduce risk of cross-infection (WHO Survey 2013).

A disposable dental blunt-ended explorer and mirror were used for each examination. The obtained information was recorded in the information form designed according to the WHO standard chart [24]. Since we did not have access to artificial light in all examinations, all the examinations were conducted in an empty classroom under natural light as recommended by the WHO oral health survey manual [24].

The oral hygiene status was determined via the Simplified Oral Hygiene Index (OHI-S) developed by Greene and Vermillion, consisting of two components of debris and calculus, each with a possible score ranges of zero to three [21]. The amounts of calculus and debris were determined by examining the facial surfaces of teeth numbers 11, 16, 26 and 31, as well as the lingual surfaces of 36 and 46. The calculus and debris scores were added and then divided by the number of examined surfaces in order to calculate the OHI-S score for each individual [21]. The CPI Index was measured in this study and was in accordance with the WHO survey manual 1997 [20]. We used a blunt explorer conformed to the periodontal probe according to the WHO specifications in the manual. Six teeth numbers of 16, 11, 26, 36, 31 and 46 were examined. Since pockets might be present because of deepened sulcus associated with eruption, only bleeding and calculus were recorded. If no index teeth or tooth was present in a sextant qualifying for examination, all the remaining teeth in that sextant are examined and the highest score is recorded as that sextant’s score. The CPI score varies in this study between 0 to 2; 0 = No periodontal disease, 1 = Bleeding on probing, 2 = Calculus with plaque seen or felt by probing [20]. A sextant is each section of the mouth defined to six sections according to tooth numbers: 14–18, 13–23, 24–28, 34–38, 33–43 and 44–48.

### Data analysis

The statistical analysis was performed using SPSS version 16.0 software. The OHI-S score varies between 0 and 6; 0–1.2 (good oral hygiene status), 1.3–3 (fair oral hygiene status) and 3.1–6 (poor oral hygiene status) [21]. In order to determine the CPI, the highest recorded score was considered as the sextant’s score and its frequency was calculated [20].

The descriptive statistics (mean, standard deviation, frequency and percentage) were calculated for all demographic variables and indicators. The logistic regression analysis was performed to examine the associations between preset independent variables (age, gender, parental occupation and education, residence district, dental pain experience in the last year as well as parental supervision while tooth brushing) with outcome variables (OHI-S and CPI indices). The 95% confidence interval (CI) and odds ratio were calculated and the significance level was considered P<0.05 in all statistical tests.

Based on previous research, the demographic profiles, including age, gender, residence district, dental pain experience, parental education and occupation, and parent supervision, could predict CPI and OHI-S [25–29].In the logistic regression analysis, it should be noted that the two clusters of unemployed fathers and Labor worker mothers due to their low frequency in the study were excluded from the modeling.

Age, gender, residence district, dental pain experience, parental education and occupation, and parental supervision were the independent/predicting variables in the Multiple and multinomial logistic regression analyses. As regards, the number of cases with poor oral hygiene was low to run the logistic regression for the OHI-S index. So, the oral hygiene status was further recoded to good oral hygiene and fair/poor oral hygiene. The fair/poor oral hygiene was calculated through combining fair and poor oral hygiene (fair oral hygiene_+_poor oral hygiene = fair/poor oral hygiene).

Multiple logistic regression analysis was executed to test the associations of preset independent/predicting variables with the outcome variable of oral hygiene status based on the calculated OHI-S. Multinomial logistic regression analysis was executed to test the associations of preset independent/predicting variables with the outcome variable of CPI including three layers of healthy, bleeding and calculus. CPI reference category is zero or healthy, the exponential estimates were expressed as odds ratios (OR) with 95% confidence intervals (CI).

## Results

The oral hygiene status was good in 644 students (65.2%), fair in 341 (34.5%) and poor in three (0.3%). Moreover, the results of CPI showed that 639 students (64.1%) had healthy periodontium, 320 (32.4%) periodontal bleeding and 29 (2.9%) were with calculus; so that 60.2% of boys and 69.3% of girls had healthy periodontium and 63.4% of boys and 67% of girls had good oral hygiene (Table 1).

**Table 1:** Distribution of OHI-S and CPI indexes among student's according to socio-demographic characteristics.

Variables	Categories	N	OHI-S^a^			CPI^b^		
			Good N (%)	Fair N (%)	Poor N (%)	Healthy N (%)	Bleeding N (%)	Calculus N (%)
gender	Boys	503	319(63.4)	183(36.4)	1(0.2)	303(60.2)	184(36.6)	16(3.2)
	Girls	485	325(67)	158(32.6)	2(0.4)	336(69.3)	136(28)	13(2.7)
Age(year)	7	153	129(84.3)	24(15.7)	00(0.0)	132(86.3)	19(12.4)	2(1.3)
	8	150	121(80.7)	29(19.3)	00(0.0)	121(80.7)	27(18)	2(1.3)
	9	158	102(64.6)	56(35.4)	00(0.0)	105(66.5)	49(31)	4(2.5)
	10	155	85(54.8)	70(45.2)	00(0.0)	87(56.1)	64(41.3)	4(2.6)
	11	178	103(57.9)	72(40.4)	3(1.7)	101(56.7)	72(40.4)	12(6.2)
	12	194	104(53.6)	90(46.4)	00(0.0)	93(47.9)	89(45.9)	12(6.2)
Residence district	Urban	571	370(64.8)	201(35.2)	00(0.0)	380(66.5)	165(28.9)	26(4.6)
	Suburban	417	274(65.7)	140(33.6)	3(0.7)	259(62.1)	155(37.2)	3(0.7)
Dental pain experience	Never	284	185(65.1)	97(34.2)	2(0.7)	178(62.7)	91(32)	15(5.3)
	Seldom	432	267(61.8)	164(38)	1(0.2)	265(61.3)	156(36.1)	11(2.5)
	Often	272	192(70.6)	80(29.4)	00(0.0)	196(72.1)	73(26.8)	3(1.1)
Father’s Education	≤Primary	58	38(65.5)	19(32.8)	1(1.7)	41(70.7)	16(27.6)	1(1.7)
	High School	790	517(65.4)	271(34.3)	2(0.3)	512(64.8)	258(32.7)	20(2.5)
	>High School	140	89(63.6)	51(36.4)	00(0.0)	86(61.4)	46(32.9)	8(5.7)
Mother’s Education	≤Primary	59	36(61.00)	22(37.30)	1(1.70)	38(64.40)	20(33.90)	1(1.70)
	High School	827	546(66.00)	279(33.70)	2(0.20)	539(65.20)	267(32.30)	21(2.50)
	>High School	102	62(60.80)	40(39.20)	00(0.00)	62(60.80)	33(32.40)	7(6.90)
Father’s occupation	Labor worker	129	82(63.6)	47(36.4)	00(0.0)	77(59.7)	47(36.4)	5(3.9)
	Government employee	631	138(61.9)	85(38.1)	00(0.0)	144(64.6)	72(32.3)	7(3.1)
	Self-employed	223	420(66.6)	208(33)	3(0.5)	415(65.8)	199(31.5)	17(2.7)
	Not employed	5	4(80)	1(20)	00(0.0)	3(60)	2(40)	00(0.0)
Mother’s occupation	Labor worker	8	5(62.5)	3(37.5)	00(0.0)	4(50)	4(50)	00(0.0)
	Government employee	101	27(45.8)	32(54.2)	00(0.0)	30(50.8)	25(42.4)	4(6.8)
	Self-employed	59	73(72.3)	28(27.7)	00(0.0)	75(74.3)	24(23.8)	2(2)
	Not employed	820	539(65.7)	278(33.9)	3(0.4)	530(64.6)	267(32.60)	23(2.8)
Parental supervision on oral hygiene	Yes	350	213(60.9)	135(38.6)	3(0.4)	210(60)	128(36.6)	12(3.4)
	No	638	431(67.6)	206(32.3)	1(0.2)	429(67.2)	192(30.1)	17(2.7)

^a^OHI-S denoted that present data for Simplified Oral Hygiene Index.

^b^CPI denoted that present data for Community Periodontal Index

Higher percentage of the boys in the age group of 12 years old had periodontal bleeding (52.9%) and fewer had good oral hygiene (51%) (Not presented in the tables).

Among the demographic factors, age (p = 0.002) and residence district (p = 0.01) were significantly associated with calculus measured by the CPI. For each year of age increase, the chance of developing calculus increased by 1.53 times. Among the independent variables, gender (p = 0.003), age (p<0.001), residence district (p = 0.01) and mother's occupation (p = 0.02) were significantly associated with gingival bleeding measured by the CPI (Table 2). For each year of age increase, the chance of developing bleeding increased by 1.44 times. Boys were 1.54 times more likely to develop gingival bleeding than girls (p = 0.003). In addition, primary school children living in the urban areas were 0.69 times less likely to develop gingival bleeding than those living in the suburbs (p = 0.01, Table 2).

**Table 2:** Association between socio-demographic factors and CPI according to multinomial logistic regression analysis.

Predictor Variables	Bleeding		Calculus	
	Adjusted OR^a^(CI 95%)	*P* value	Adjusted OR^a^(CI 95%)	*P* value
**Age** (year)	1.44(1.31–1.59)	<0.001	1.53(1.17–2.02)	0.002
**Gender**				
Boys	1.54 (1.16–2.06)	0.003	1.43(0.65–3.11)	0.36
Girls(Reference category)				
**Residence district**				
Urban	0.69 (0.51–0.93)	0.01	5.21(1.49–18.18)	0.01
Suburban(Reference category)				
**Dental Pain Experience**				
Never	0.97 (0.64–1.45)	0.88	2.97(0.79–11.18)	0.10
Seldom	1.12(0.78–1.61)	0.53	1.58(0.41–6.11)	0.50
Often(Reference category)				
**Father’s Education**				
≤Primary	0.52 (0.19–1.42)	0.20	0.48(0.02–8.25)	0.61
High School	0.88(0.51–1.51)	0.65	0.73(0.22–2.40)	0.60
>High School(Reference category)				
**Mother’s Education**				
≤Primary	1.43(0.51–4.01)	0.48	0.60(0.03–11.04)	0.73
High School	1.05(0.55–1.99)	0.87	0.59(0.15–2.20)	0.43
>High School(Reference category)				
**Father’s Occupation**				
Labor worker	1.50(0.89–2.55)	0.12	3.63(0.91–14.44)	0.06
Self-employed	1.13(0.78–1.64)	0.50	1.63(0.59–4.46)	0.34
Government employee(Reference category)				
**Mother’s Occupation**				
Not employed	0.55(0.29–1.05)	0.07	0.61(0.16–2.36)	0.48
Self-employed	0.40(0.18–0.86)	0.02	0.42(0.06–2.81)	0.37
Government employee(Reference category)				
**Parental supervision on oral hygiene**				
Yes	0.85 (0.62–1.17)	0.32	0.78(0.34–1.78)	0.55
No(Reference category)				

CPI reference category is: healthy

CI, confidence interval; OR, odds ratio

^a^The regression was adjusted for sex & age

Age (p<0.001) and mother's occupation (p = 0.008) were significantly associated with OHI-S index. For each year of age increase, the likelihood of developing poor and fair oral hygiene increased 1.36 times (p<0.001). The primary school children with mothers employed in the government were 2.27 more likely to have fair/poor oral hygiene than those with unemployed or self-employed mothers (Table 3, p = 0.008).

**Table 3:** Association between socio-demographic factors and OHI-S according to multiple logistic regression analysis.

Predictor Variables	Adjusted OR^a^ (CI 95%)	*P* value
**Age** (year)	1.36(1.24–1.48)	<0.001
**Gender**		
Boys(Reference category)		
Girls	0.85(0.64–1.11)	0.24
**District**		
Center (Reference category)		
Suburb	0.96(0.71–1.28)	0.79
**Dental Pain Experience**		
Never (Reference category)		
Seldom	0.86(0.63–1.18)	0.36
Often	1.28(0.89–1.83)	0.17
**Father’s Education**		
≤Primary (Reference category)		
High School	1.18(0.53–2.58)	0.67
>High School	1.00(0.39–2.55)	0.99
**Mother’s Education**		
≤Primary (Reference category)		
High School	0.66(0.31–1.41)	0.29
>High School	0.66(0.25–1.77)	0.41
**Father’s Occupation**		
Labor worker (Reference category)		
Self-employed	0.91(0.59–1.39)	0.66
Government employee	0.97(0.58–1.60)	0.91
**Mother’s Occupation**		
Not employed (Reference category)		
Self-employed	0.81(0.52–1.29)	0.39
Government employee	2.27(1.23–4.17)	0.008
**Parental supervision on oral hygiene**		
Yes (Reference category)		
No	1.12(0.83–1.52)	0.43

*Dependent* variable, OHI-S, was dichotomized as good = 0, fair/poor oral hygiene = 1

CI, confidence interval; OR, odds ratio

^a^The regression was adjusted for sex & age

## Discussion

The results of this study showed that the highest prevalence of periodontal problem was observed in the boys aged 12 years of age since 52.9% had gingival bleeding according to CPI scores and the fair oral hygiene was 49%. By comparing the present study’s results with other studies, it was found that the gingival bleeding component of CPI in this study was 1.42 times higher in 7 year old students and also 3.61 times higher in the 12 years old age group when compared with the same age groups in the 2012 national survey results for the Hamadan population [12]. The comparison of results with similar studies shows that the prevalence of bleeding and calculus in the study participants was lower than similar age groups in other studies[30–33]. The healthy CPI obtained from this study was similar to the results of studies conducted in Bosnia and Herzegovina (CPI score of 0 = 43%) [34], and better than Chile (CPI score 0 = 31.58%), India (CPI score 0 = 14.10), in China among Bulang people (CPI score 0 = 29%) and Yunnan participants (CPI score 0 = 7%), and Burkina Faso (CPI score 0 = 22%) [30–33, 35]. In general, the differences in CPI reports in various studies may be related to the changes in geographic locations of studies, variations in age, demographics and socio-economic characteristics of the study population.

The results of this study also showed that the mean OHI-S scores of students was approximately similar to OHI-S scores reported of students in India and other studies in Iran[36–40] and better than the OHI-S scores reported of students in Nigeria, Thailand, Vietnam, Peru and some cities in India[25, 41–45] and worse than Greece and Portugal[46–48]. Studies indicate that various variables, including conducting preventive programs, educating oral hygiene behaviors, changing attitude of families and dentists towards oral health in a positive manner, the provision of affordable, as well as acceptable and high-quality oral healthcare services all can affect gingival and periodontal health[16].The general dental services such as preventive interventions (fluoride therapy and fissure sealants), comprehensive prevention program through oral hygiene education by involving parents and school staff in the process of training and application of fluoride for children in countries such as Greece and Portugal has led to the improvement of the oral hygiene and periodontal health of the students since 1980 [46–48].

Dental care should be started according to the recommendation of the American Academy of Pediatric Dental Association from the infant age[49]. Due to the lack of cognitive and functional skills in young children for performing oral hygiene behaviors, children are provided oral care by their parents or guardians [50]. It is recommended that parents at least help them with their routine brushing and flossing until the child reaches school age and becomes 7 years old. Therefore, educating parents will increase their knowledge and skills and improve their attitude, which in turn will improve, and affect children’s oral hygiene habits and behaviors. Since the transition from primary to permanent tooth takes place in the early years of school age, educating parents at this period can be considered critical. However, the oral health has been ignored for a long time in Iran due to various causes such as inappropriate attitude, inadequate knowledge on oral health, oral health care not being a priority and the existence of social and economic inequalities [13–15]. In Iran, the national project for oral health promotion which is based on education, clinical examination, determination of the treatment needs, completion of the electronic individual oral health records and provision of prevention services (using fluoride varnish) for primary school students has been recently approved in 2015.However, the program is limited to the use of fluoride varnish and there is still a great gap in education. In addition, high costs and the lack of proper dental insurance and access to dental services in most parts of the country have caused Iranian children to be deprived of proper oral health [13–15].

Like most studies, the present study’s findings showed that the prevalence of teeth with periodontal bleeding, presence of calculus and the oral hygiene index were related to age, so that younger children had a better oral hygiene status and periodontal health[25, 26, 41, 51–54]. Studies indicate that the prevalence and development of periodontal diseases increase with age, and has been initiated since the age of tooth eruption and reaches its peak at puberty [55]. In this study, the results of CPI indicated that for each year of age increase, the bleeding increased by 1.14 folds, the calculus by 1.53 folds and the odds of poor/fair oral hygiene status increased by 1.36 folds. One of the reasons for this is the cumulative effect of plaque and calculus with age, which results in the gingival bleeding and poor oral hygiene [56–59].Inflammation of the gingival is a response to the bacterial plaque build at and below the gingival margin [60, 61].Individuals with good oral hygiene behavior and regular flossing followed by brushing can disrupt the accumulation of dental plaque and eventually prevent gingivitis[62]. This can also be due to the presence of mixed dentition, various dietary habits, primary tooth exfoliation, puberty and inappropriate oral hygiene[60, 63].

The results of CPI depicted that gender is associated with the gingival bleeding score. The incidence of bleeding in boys was 1.54 times more likely than that in girls. Al-Haddad et al.[64], Tomazoni et al.[27], Zhang et al.[33] and Kumar et al.[65] have also reported the same results. This can be due to the fact that the boys are less interested in adhering to the oral hygiene behaviors and recommendations than the girls are, and the girls usually have a better oral hygiene level and adherence to brushing more regularly than the boys[37, 66, 67]. Nevertheless, the current results are different from the studies from Udaipur in India [63], Sharjah in United Arab Emirates [26] and Riydh in Saudi Arabia [68]. They stated that the periodontal health status in the boys is better than in girls. This disagreement can be due to the cultural differences since in these countries, parents pay more attention to their sons and prioritize their needs compared to their daughters [26].

Similar to some studies, our findings show that the place of residence is associated with the number of sextants with periodontal bleeding and calculus[28, 53, 69–71]. Primary school students living in the suburbs are 1.44 times more likely to have bleeding in their gums compared to urban students. The effect of the residence on oral health can be justified with different mechanisms. Generally, people living in suburban areas have lower socioeconomic status; hence, their lifestyle behaviors are less healthy and high-risk due to poor economic resources and inability to choose healthy options which all can affect their oral hygiene [27, 72]. On the other hand, the oral health status depends on the frequency, type and quality of tooth brushing. The oral hygiene status among suburban students can be attributed to poor oral hygiene, not brushing or flossing and brushing or flossing infrequently. Limited access to professional dental care, restorative dentistry or any kind of dental treatment also influence oral health status [13, 15, 36, 73]. In this study, the odds ratio of calculus in urban schoolchildren was 5.21 times higher than that of suburban schoolchildren, which is different from the results of other studies[53, 69–71]. This inconsistency was due to the small sample size of primary school students with calculus, so that only 29 out of 988 students had calculus.

The results of this study showed that mother's occupation is related to the number of sextants with periodontal bleeding and oral hygiene status. Qajari et al., Zurriaga et al., and Sim et al., also reported the same results[29, 74, 75]. In this study, the children with self-employed mothers were 0.40 times less likely to develop gingival bleeding than those with government-employed mothers. The OHI-S scores of children whose mothers were employed in the government showed that they were 2.27 times more likely to have fair/poor oral hygiene than those with non-employed mothers. This may be because in Iran, mothers employed in governmental sectors are working from 7:30 am to 2:00 pm and sometimes up to 5:00 pm. Therefore, when they get home they get busy with the household chores such as cooking, shopping and cleaning. They have less time to spend on the oral hygiene of their children compared to housewives and women who are self-employed.

Many studies, including our research have shown that parental education and fathers’ occupation do not predict the gingival bleeding, calculus and oral hygiene in their children[76–81]. However, the signs and symptoms of periodontal disease are highly associated with age and are usually observed later in life which might be another reason for this inconsistency in results[82]. In a study by Ayo-Yusuf et al., it has been determined that specific psychological features such as the degree of children’s dependency to their parents, can affect the relationship between parental socioeconomic characteristics and children’s oral health [83]. The socioeconomic characteristics of parents can indirectly affect their children's oral health through their psychological status and mental health such as life satisfaction, stress and perceived control, and coping styles among social groups[72, 84]. In addition, people in the early adolescence are strongly opinionated and often object to their parents' recommendations, affecting their oral health status [77].

This study had some limitations. First, the cross-sectional nature of the study does not show causal relationship between the students’ gingivitis, oral hygiene status and their socio-demographic characteristics. However, cross-sectional studies are important in identifying risk factors, which could be used for maintaining population health and conducting future cohort or longitudinal assessments based on the identified risk factors.

In this study, the CPI was used to investigate the prevalence and severity of periodontal disease which has many criticisms that are based on the progressive definition of periodontal disease; so that a tooth with a pocket present must also have calculus and bleeding[85, 86]. In addition, the examination of the indicated teeth compared to the full-mouth examination might underestimate the true prevalence of the periodontal problem [87]. However, the use of this index is easy and it is the main source of epidemiological information on the periodontal disease in many countries. Some international organizations, such as WHO, recommend the CPI to assess the prevalence and development of the gingivitis in the population [88]; so that the possible international comparisons could be made.

The statistical results in this study could also be affected by socially acceptable responses about parent's occupation, educational level and positive supervision on primary school aged children’s tooth brushing. This might suggest that socioeconomic characteristics of the parents could not predict the gingivitis and the oral hygiene status in primary school students[30, 89, 90]. However, it should be noted that we addressed this limitation by reviewing the schools’ record books, which are developed for each student and their parents’ occupation in all primary schools. We did not found any discrepancy between self-reported data and school records.

One of the strengths of this study is the large sample size of 7–12 years old primary school students in Hamadan for assessing the oral hygiene status and periodontal health compared to similar studies[25, 41, 43, 78, 80, 91]. This sample was selected by a three-stage random sampling design, which included students from public and private schools in different parts of the city. The sampling precision and high power in generalizability of this study are from its strengths. Additionally, the high response rate from students and one single examiner increased the internal validity. Another important advantage of this research is that we did not focus solely on biological aspects, but also studied socio-demographic methods, which are part of the overall picture of the natural history of oral diseases in the epidemiological studies. Despite the potential mentioned limitations, this study can contribute to the epidemiological studies of gingivitis and the oral hygiene level in children and adolescents, by providing a better understanding of the dynamic process of the oral hygiene. Our findings can help design, implement wide scale interventional health promotion programs, and oversee local dental strategies for better access to dental care.

## Conclusion

In general, the study results demonstrated that more than 60% of the Hamadan primary school students had healthy gingiva and periodontium (64.1%) and their oral hygiene status was good (65.2%). Age, gender, residence district and mother’s occupation were significantly associated with bleeding and calculus components of the CPI. Furthermore, age and mother's occupation were significantly associated with the oral hygiene index. More children from urban areas had healthy periodontium than those living in suburban areas and boys compared to girls. The current results indicate that oral hygiene is an important public health concern among 7–12 years old students in Hamadan primary schools. Therefore, an active and effective preventive program is essential for improving pediatric oral hygiene status, especially for children attending to suburban schools.

## Supporting information

S1 Table Mean score s and standard deviation s by age group for debris, Calculus, OHI-S and CPI. (DOC)

S2 Table Distribution of OHI-S and CPI indexes among student's by age group according to sociodemographic characteristics (mean±SD). (DOC)

S1 Dataset Statistics of participant. (SAV)
