# Supplementary material for: Socio-demographic determinants as predictors of oral hygiene status and gingivitis in schoolchildren aged 7-12 years old: A cross-sectional study
Source: PLoS One. 2018 Dec 14;13(12):e0208886. doi: 10.1371/journal.pone.0208886 (PMC6294426; doi:10.1371/journal.pone.0208886)
Supplement: S1 Table — (DOC) [file pone.0208886.s001.doc]

Table 1 • Mean score s and standard deviation s by age group for debris, Calculus, OHI-S and CPI

| Age 12 (n=194) |  | Age 11 (n=178) |  | Age 10 (n=155) |  | Age 9 (n=158) |  | Age 8 (n=150) |  | Age 7 (n=153) | variables |
| --- | --- | --- | --- | --- | --- | --- | --- | --- | --- | --- | --- |
| Mean (SD) |  | Mean (SD) |  | Mean (SD) |  | Mean (SD) |  | Mean (SD) |  | Mean (SD) |
| 1.25(0.54) |  | 1.20(0.56) |  | 1.29(0.56) |  | 1.20(0.50) |  | 1.00(0.51) |  | 0.92(.049) | Debris |
| 0.01(0.08) |  | 0.02(0.21) |  | 0.02(0.10) |  | 0.002(0.02) |  | 0.01(0.10) |  | 0.005(0.04) | Calculus |
| 1.27(0.53) |  | 1.23(0.63) |  | 1.31(0.57) |  | 1.20 (0.50) |  | 1.01 (0.50) |  | 0.93(0.49) | OHI-S^a^ |
| 0.58(0.60) |  | 0.46(0.55) |  | 0.46(0.54) |  | 0.36 (0.53) |  | 0.20(0.43) |  | 0.15(0.39) | CPI^b^ |

^a^OHI-S denoted that present data for Simplified Oral Hygiene Index.

^b^CPI denoted that present data for Community Periodontal Index
