# Supplementary material for: Socio-demographic determinants as predictors of oral hygiene status and gingivitis in schoolchildren aged 7-12 years old: A cross-sectional study
Source: PLoS One. 2018 Dec 14;13(12):e0208886. doi: 10.1371/journal.pone.0208886 (PMC6294426; doi:10.1371/journal.pone.0208886)
Supplement: S2 Table — (DOC) [file pone.0208886.s002.doc]

Table 2 •Distribution of OHI-S and CPI indexes among student's by age group according to sociodemographic characteristics (mean±SD).

| Age 12 | |  | Age 11 | |  | Age 10 | |  | Age 9 | |  | Age 8 | |  | Age 7 | |  |  |
| --- | --- | --- | --- | --- | --- | --- | --- | --- | --- | --- | --- | --- | --- | --- | --- | --- | --- | --- |
| OHI-S | CPI |  | OHI-S | CPI |  | OHI-S | CPI |  | OHI-S | CPI |  | OHI-S | CPI |  | OHI-S | CPI |  |  |
| Mean (SD) | Mean (SD) |  | Mean(SD) | Mean (SD) |  | Mean (SD) | Mean (SD) |  | Mean (SD( | Mean (SD) |  | Mean (SD) | Mean (SD) |  | Mean (SD) | Mean (SD) | Categories | variables |
| 1.30(0.49) | 0.66(0.60) | 1.27(0.58) | | 0.46(0.54) | 1.39(0.62) | | 0.52(0.55) | 0.39(0.81) | | 4.99(2.85) | 0.94 (0.52) | | 0.19(0.39) | 0.97(0.55) | | 0.20(0.46) | Boy | gender |
| 1.24(0.57) | 0.48(0.60) | 1.19(0.67) | | 0.46(0.56) | 1.24(0.50) | | 0.40(0.54) | 1.12(1.58) | | 4.83(2.71) | 1.09 (0.47) | | 0.22(0.48) | 0.89(0.42) | | 0.09(0.29) | Girl |
| 1.26(0.55) | 0.62(0.66) | 1.18(0.55) | | 0.39(0.55) | 1.31(0.57) | | 0.50(0.59) | 0.82(1.31) | | 5.20(2.87) | 0.97(0.53) | | 0.18(0.44) | 0.91(0.52) | | 0.13(0.37) | Urban | District |
| 1.30(0.50) | 0.52(0.50) | 1.29(0.70) | | 0.53(0.54) | 1.31(0.57) | | 0.42(0.49) | 0.68(1.32) | | 4.42(2.55) | 1.07(0.44) | | 0.23(0.42) | 0.95(0.45) | | 0.16(0.41) | Suburban |
| 1.19(0.55) | 0.58(0.67) | 1.28(0.65) | | 0.45(0.55) | 1.29(0.56) | | 0.55(0.58) | 0.81(1.14) | | 3.93(2.98) | 0.94(0.59) | | 0.18(0.46) | 1.04(0.43) | | 0.13(0.41) | Never | Dental pain experience |
| 1.29(0.52) | 0.57(0.56) | 1.20(0.63) | | 0.47(0.57) | 1.36(0.61) | | 0.49(0.55) | 0.80(1.51) | | 4.62(2.65) | 0.99(0.53) | | 0.24(0.47) | 0.89(0.46) | | 0.22(0.46) | Seldom |
| 1.48(0.46) | 0.66(0.59) | 1.21(0.57) | | 0.44(0.50) | 1.23(0.49) | | 0.29(0.46) | 0.71(1.20) | | 5.53(2.68) | 1.07(0.40) | | 0.18(0.39) | 0.89(0.55) | | 0.08(0.28) | Often |
| 1.35(0.51) | 0.28(0.48) | 1.66(0.96) | | 0.88(0.60) | 1.05(0.51) | | 0.22(0.44) | 0.79(1.36) | | 4.57(1.95) | 0.89(0.26) | | 0.09(0.30) | 1.12(0.40) | | 0.12(0.35) | ≤Primary | Father’s education |
| 1.24(0.53) | 0.57(0.60) | 1.22(0.60) | | 0.41(0.50) | 1.33(0.56) | | 0.48(0.54) | 0.80(1.36) | | 5.06(2.93) | 1.03(0.52) | | 0.24(0.47) | 0.93(0.50) | | 0.15(0.40) | High School |
| 1.41(0.53) | 0.68(0.63) | 10.16(0.0) | | 0.57(0.69) | 1.34(0.61) | | 0.47(0.61) | 0.57(0.97) | | 4.24(2.18) | 1.01(0.51) | | 0.10(0.05) | 0.72(0.12) | | 0.12(0.12) | >High School |
| 1.45(0.52) | 0.50(0.52) | 1.95(0.82) | | 0.85(0.69) | 1.18(0.44) | | 0.25(0.46) | 0.60(1.29) | | 4.80(1.65) | 1.00(0.24) | | 0.18(0.40) | 1.02(0.22) | | 0.12(0.35) | ≤Primary | Mother’s education |
| 1.26(0.52) | 0.56(0.59) | 1.21(0.61) | | 0.43(0.52) | 1.30(0.57) | | 0.46(0.54) | 0.80(1.35) | | 5.00(2.95) | 1.03(0.51) | | 0.21(0.45) | 0.94(0.51) | | 0.15(0.40) | High School |
| 1.29(0.61) | 0.72(0.70) | 1.13(0.57) | | 0.54(0.67) | 1.45(0.60) | | 0.60(0.63) | 0.60(0.98) | | 4.20(1.97) | 0.93(0.54) | | 0.15(0.36) | 0.68(0.18) | | 0.12(0.35) | >High School |
| 1.41(0.57) | 0.64(0.55) | 1.23(0.58) | | 0.47(0.62) | 1.32(0.45) | | 0.59(0.59) | 0.37(0.95) | | 5.47(2.48) | 1.09(0.46) | | 0.16(0.38) | 0.90(0.46) | | 0.16(0.37) | worker | Father’s job |
| 1.21(0.49) | 0.56(0.62) | 1.22(0.65) | | 0.47(0.56) | 1.30(0.57) | | 0.45(0.55) | 0.76(1.20) | | 5.10(2.72) | 0.99(0.46) | | 0.18(0.41) | 0.93(0.47) | | 0.14(0.38) | Self-employment |
| 1.31(0.57) | 0.57(0.61) | 1.27(0.59) | | 0.40(0.49) | 1.33(0.65) | | 0.41(0.50) | 0.97(1.70) | | 4.16(2.96) | 1.03(0.67) | | 0.30(0.54) | 0.97(0.57) | | 0.16(0.45) | Government service |
| 1.50(0.70) | 1.00(0.00) | 0.00 | | 0.00 | 0.00 | | 0.00 | 1.00(1.41) | | 3.50(3.53) | 0.00 | | 0.00 | 0.00 | | 0.00 | No job |
| 1.00(0.00) | 1.00(0.00) | 0.00 | | 0.00 | 0.00 | | 0.00 | 1.00(1.73) | | 6.80(2.16) | 0.00 | | 0.00 | 1.33(0.94) | | 0.50(0.70) | worker | Mother’s job |
| 1.08(0.41) | 0.18(0.40) | 0.87(0.33) | | 0.29(0.46) | 1.25(0.56) | | 0.50(0.60) | 1.00(1.75) | | 4.31(3.19) | 0.98(0.62) | | 0.13(0.35) | 0.92(0.33) | | 0.11(0.33) | Self-employment |
| 1.42(0.67) | 0.84(0.68) | 1.20(0.59) | | 0.56(0.62) | 1.81(0.60) | | 0.75(0.46) | 0.73(1.10) | | 4.45(2.42) | 1.33(0.81)) | | 0.25(0.50) | 1.04(0.48) | | 0.28(0.48) | Government service |
| 1.28(0.53) | 0.59(0.60) | 1.27(0.65) | | 0.46(0.55) | 1.29(0.55) | | 0.44(0.54) | 0.73(1.26) | | 4.94(2.76) | 0.98(0.62) | | 0.13(0.35) | 1.33(0.94) | | 0.14(0.39) | No job |
| 1.30(0.55) | 0.54(0.58) | 1.22(0.67) | | 0.43(0.54) | 1.25(0.50) | | 0.45(0.57) | 1.19(1.57) | | 5.06(2.83) | 1.02(0.52) | | 0.15(0.36) | 1.03(0.81) | | 0.06(0.25) | Yes | Parent supervision |
| 1.23(0.50) | 0.63(0.64) | 1.24(0.58) | | 0.48(0.56) | 1.37(0.61) | | 0.47(0.52) | 0.66(1.22) | | 4.87(2.77) | 1.01(0.50) | | 0.21(0.45) | 0.92(0.44) | | 0.15(0.40) | No |
